# Integrated multi‐omics approach reveals the role of striated muscle preferentially expressed protein kinase in skeletal muscle including its relationship with myospryn complex

**DOI:** 10.1002/jcsm.13470

**Published:** 2024-05-09

**Authors:** Qifei Li, Jasmine Lin, Shiyu Luo, Klaus Schmitz‐Abe, Rohan Agrawal, Melissa Meng, Behzad Moghadaszadeh, Alan H. Beggs, Xiaoli Liu, Mark A. Perrella, Pankaj B. Agrawal

**Affiliations:** ^1^ Division of Neonatology, Department of Pediatrics University of Miami Miller School of Medicine and Holtz Children's Hospital, Jackson Health System Miami FL USA; ^2^ Division of Genetics and Genomics Boston Children's Hospital, Harvard Medical School Boston MA USA; ^3^ The Manton Center for Orphan Disease Research Boston Children's Hospital, Harvard Medical School Boston MA USA; ^4^ Division of Pulmonary and Critical Care Medicine Brigham and Women's Hospital, Harvard Medical School Boston MA USA; ^5^ Department of Pediatric Newborn Medicine Brigham and Women's Hospital, Harvard Medical School Boston MA USA

**Keywords:** congenital myopathy, multi‐omics, myospryn complex, skeletal muscle, striated muscle preferentially expressed protein kinase

## Abstract

**Background:**

Autosomal‐recessive mutations in *SPEG* (striated muscle preferentially expressed protein kinase) have been linked to centronuclear myopathy with or without dilated cardiomyopathy (CNM5). Loss of SPEG is associated with defective triad formation, abnormal excitation–contraction coupling, calcium mishandling and disruption of the focal adhesion complex in skeletal muscles. To elucidate the underlying molecular pathways, we have utilized multi‐omics tools and analysis to obtain a comprehensive view of the complex biological processes and molecular functions.

**Methods:**

Skeletal muscles from 2‐month‐old SPEG‐deficient (*Speg*‐CKO) and wild‐type (WT) mice were used for RNA sequencing (*n* = 4 per genotype) to profile transcriptomics and mass spectrometry (*n* = 4 for WT; *n* = 3 for *Speg*‐CKO mice) to profile proteomics and phosphoproteomics. In addition, interactomics was performed using the SPEG antibody on pooled muscle lysates (quadriceps, gastrocnemius and triceps) from WT and *Speg*‐CKO mice. Based on the multi‐omics results, we performed quantitative real‐time PCR, co‐immunoprecipitation and immunoblot to verify the findings.

**Results:**

We identified that SPEG interacts with myospryn complex proteins CMYA5, FSD2 and RyR1, which are critical for triad formation, and that SPEG deficiency results in myospryn complex abnormalities (protein levels decreased to 22 ± 3% for CMYA5 [*P* < 0.05] and 18 ± 3% for FSD2 [*P* < 0.01]). Furthermore, SPEG phosphorylates RyR1 at S2902 (phosphorylation level decreased to 55 ± 15% at S2902 in *Speg*‐CKO mice; *P* < 0.05), and its loss affects JPH2 phosphorylation at multiple sites (increased phosphorylation at T161 [1.90 ± 0.24‐fold], S162 [1.61 ± 0.37‐fold] and S165 [1.66 ± 0.13‐fold]; decreased phosphorylation at S228 and S231 [39 ± 6%], S234 [50 ± 12%], S593 [48 ± 3%] and S613 [66 ± 10%]; *P* < 0.05 for S162 and *P* < 0.01 for other sites). On analysing the transcriptome, the most dysregulated pathways affected by SPEG deficiency included extracellular matrix–receptor interaction (*P* < 1e^−15^) and peroxisome proliferator‐activated receptor signalling (*P* < 9e^−14^).

**Conclusions:**

We have elucidated the critical role of SPEG in the triad as it works closely with myospryn complex proteins (CMYA5, FSD2 and RyR1), it regulates phosphorylation levels of various residues in JPH2 and S2902 in RyR1, and its deficiency is associated with dysregulation of several pathways. The study identifies unique SPEG‐interacting proteins and their phosphorylation functions and emphasizes the importance of using a multi‐omics approach to comprehensively evaluate the molecular function of proteins involved in various genetic disorders.

## Introduction

Congenital myopathies (CMs) present with skeletal muscle weakness and hypotonia of varying severity, time of onset and genetic cause and are often subclassified by pathological findings. Centronuclear myopathy (CNM) is a common subtype characterized by the mislocalization of nuclei to the centre of myofibre instead of the periphery. The clinical spectrum of CNM is diverse among affected individuals, and ~60–80% of CNM can be explained by mutations in myotubularin 1 (*MTM1*),^1^ dynamin 2 (*DNM2*),^2^ bridging integrator 1 (*BIN1*),^3^ ryanodine receptor 1 (*RyR1*),^4^ voltage‐dependent L‐type calcium channel subunit alpha‐1S (*CACNA1S*)^5^ and striated muscle preferentially expressed protein kinase (*SPEG*).^6^

We have previously linked recessive variants in *SPEG* with CNM, CM or dilated cardiomyopathy (DCM), CNM and DCM often presenting together.^6^, ^7^ SPEG is a member of the myosin light chain kinase (MLCK) protein family, important for myocyte function and the regulation of the actin‐based cytoskeleton.^8^ Using wild‐type (WT) mouse model, we have determined that SPEG localizes in a double line, in alignment with the terminal cisternae of the sarcoplasmic reticulum (SR) of the triad.^9^ Skeletal excitation–contraction (E–C) coupling and calcium homeostasis require triads, the nanoscopic microdomains formed adjacent to Z lines by apposition of transverse tubules and terminal cisternae of the SR.^10^ The constitutive *Speg*‐knockout (KO) mice die in utero or shortly after birth due to heart failure,^11^ and to overcome the perinatal lethality, we created floxed *Speg* mice and crossed them with muscle creatine kinase (*Mck*) cre‐expressing mice, which give rise to striated muscle‐specific *Speg*‐KO (*Speg*‐CKO) mice.^9^ The late onset of cre expression in *Mck*‐cre mice (starts at Embryonic Day 17, peaks at Postnatal Day 10 and remains high thereafter) prevents early death as the *Speg*‐CKO mice can live for a few months.^9^ Using *Speg*‐CKO mice, we have shown that SPEG deficiency is associated with uniquely defective triad formation leading to abnormal E–C coupling and calcium mishandling in skeletal muscles.^9^ Triad abnormalities are also seen with DNM2,^12^ MTM1^13^ and BIN1^14^ proteins that are associated with CNM, and we have previously demonstrated that SPEG interacts with MTM1^6^ and DNM2.^15^ The molecular relationship of SPEG with triadic proteins and the effects of SPEG deficiency on downstream pathways need to be elucidated.

Here, we utilized the *Speg*‐CKO mouse model and applied multi‐omics approaches (interactomic, proteomic, phosphoproteomic and transcriptomic analyses) to determine SPEG‐interacting proteins and proteins and molecular pathways affected by SPEG deficiency. We identified members of the myospryn complex that include cardiomyopathy‐associated protein 5 (CMYA5 or myospryn), fibronectin type III and SPRY domain‐containing protein 2 (FSD2 or minispryn), and ryanodine receptor 1 (RyR1) as novel SPEG‐interacting proteins using mass spectrometry (MS)‐based protein–protein interaction analysis and confirmed by co‐immunoprecipitation (co‐IP) assays. In addition, SPEG phosphorylates RyR1 (S2902 in particular) and multiple sites of JPH2. Furthermore, SPEG deficiency is associated with a marked reduction in CMYA5, FSD2 and MTM1 protein levels. Moreover, transcriptomic analysis indicates dysregulated pathways of extracellular matrix (ECM)–receptor interaction suggestive of defective focal adhesion. Mitochondrial abnormalities due to SPEG deficiency were also noted on electron microscopy (EM) and gene set enrichment analysis (GSEA) of proteomic data.

## Methods

### Animal model

All studies were approved by the Institutional Animal Care and Use Committee at Boston Children's Hospital (Approval Number 20‐05‐4179). The work followed the Guide for the Care and Use of Laboratory Animals and all the regulatory protocols set forth by the Boston Children's Hospital Animal Resources at Children's Hospital (ARCH) facility. Homozygous *Speg*‐conditional KO mice (*Speg* ^*fl/fl*^) were generated as previously described and bred with male transgenic mice who have the Cre recombinase driven by muscle creatine kinase promoter (*MCK‐Cre* ^*+*^), with Cre activity observed in skeletal and cardiac muscle.^9^ *Speg*‐CKO (*Speg* ^*fl/fl*^ */MCK‐Cre* ^*+*^) and WT (*Speg* ^*fl/fl*^ */MCK‐Cre* ^*−*^ or *Speg* ^*fl/+*^ */MCK‐Cre* ^*−*^) mice were used in this study. Specific primers were designed to identify *Speg* ^*fl/fl*^ and *MCK‐Cre* ^*+*^ alleles.^9^ In the figure legends, each experiment's sample size is specified.

### Immunoprecipitation to determine SPEG interactome

Immunoprecipitation of SPEG was performed on pooled skeletal muscles (quadriceps, gastrocnemius and triceps) from at least three WT and *Speg*‐CKO mice for the experiment. A rabbit anti‐SPEG antibody (12472‐T16, 1:50 dilution, Sino Biological, Beijing, China) was used for immunoprecipitation. Samples were concentrated and analysed by sodium dodecyl sulfate–polyacrylamide gel electrophoresis (SDS–PAGE) and stained with Coomassie Blue (1610786, Bio‐Rad Laboratories, Hercules, CA, USA). The IgG control and the *Speg*‐CKO lysate incubated with SPEG antibody were used as controls. Experimental details are described in the supporting information. After peptide matching using Sequest (Thermo Fisher Scientific, Waltham, MA, USA),^16^ the list of identified proteins was analysed using the following filtering criteria: (1) detected only in WT muscle after SPEG IP, (2) detected with at least four unique peptides, (3) absent in IgG IP and (4) absent in *Speg*‐CKO muscle after SPEG IP.

### Co‐immunoprecipitation

Skeletal muscle lysates from quadriceps, gastrocnemius and triceps were obtained by homogenization using CryoGrinder and lysed in Pierce IP lysis buffer (PI87787, Thermo Fisher Scientific) supplemented with Halt Protease and Phosphatase Inhibitor (PI78441, Thermo Fisher Scientific) and 5‐mM ethylenediaminetetraacetic acid (EDTA) (final concentration). The tissue was lysed at 4°C for 30 min. After centrifugation (16 000 *g*, 4°C, 20 min), the soluble fractions were collected, and the concentration was measured using a colorimetric bicinchoninic acid (BCA) assay (23225; Thermo Fisher Scientific). Soluble homogenates were precleared with Dynabead Protein G beads (Thermo Fisher Scientific) for 1 h, and supernatants were incubated with specific antibodies directed against the protein of interest at 4°C for 12–24 h. Dynabead Protein G beads were then added for 2 h to capture the immune complex. The beads were washed three times with co‐IP buffer supplemented with 0.1% CHAPS. For all experiments, two negative controls consisted of a sample lacking the primary antibody and a sample incubated with another primary antibody from the same serotype as the antibody of interest. The resulting beads were eluted with Laemmli buffer and subjected to SDS–PAGE followed by immunoblotting. Protein isolation and western blot (WB) procedures were performed as described previously.^17^ The antibodies used in the experiments are listed in the supporting information.

### Whole proteomic, phosphoproteomic and transcriptomic profiling

For proteomic and phosphoproteomic analyses, quadriceps femoris samples of 2‐month‐old WT (*n* = 4) and *Speg*‐CKO (*n* = 3) mice were lysed and processed as described in the SPEED protocol.^18^ For transcriptomic analysis, quadriceps femoris samples were isolated from WT and *Speg*‐CKO mice (*n* = 4 per group). Experiment details were described in the supporting information.

### Bioinformatic analysis of omics data

For proteomic and transcriptomic analyses, differentially expressed proteins (DEPs) and differentially expressed genes (DEGs) were both analysed for Gene Ontology (GO) and Kyoto Encyclopedia of Genes and Genomes (KEGG) using g:Profiler (Version e107_eg54_p17_bf42210).^19^ Additionally, proteomic data were subjected to unbiased GSEA, and all GSEA plots including the GSEA enrichment plot and enrichment map were generated with the GSEA software (Version 4.2.1).^20^ The enrichment map was exported with Cytoscape (Version 3.9.1).^21^ Plots of KEGG pathways were generated using KEGG Mapper (Version 5.0).^22^

### Transmission electron microscopy

Skeletal muscle samples of WT and *Speg*‐CKO quadriceps (1‐ to 2‐mm cubes, *n* = 3 per group) were fixed in 2.5% glutaraldehyde, 1.25% paraformaldehyde and 0.03% picric acid in 0.1‐M sodium cacodylate buffer (pH 7.4) overnight at room temperature and stored at 4°C. Sample processing and EM were performed as described previously.^17^ These were performed at the EM Core of Harvard Medical School.

### Statistical analysis

Results were analysed with GraphPad Prism (Version 8.0; GraphPad Software) and expressed as mean ± standard deviation (SD). An unpaired two‐tailed *t* test was used to determine statistically significant differences for two‐group comparisons. One‐way analysis of variance (ANOVA) followed by Tukey's post hoc test was used for multiple‐group comparisons. The numbers of samples per group (*n*) and statistical significance for all comparisons are specified in the figure legends. *P* < 0.05 was considered statistically significant.

### Data availability

The MS data (interactomic, proteomic and phosphoproteomic) have been deposited to the ProteomeXchange Consortium via the PRIDE partner repository with the dataset identifier PXD041692. RNA‐sequencing (RNA‐seq) data were deposited in the National Center for Biotechnology Information (NCBI) Sequence Read Archive (SRA): SUB12947820.

The supporting information for this article is available online.

## Results

### Levels of SPEGα and SPEGβ increase over time

**Figure 1:**
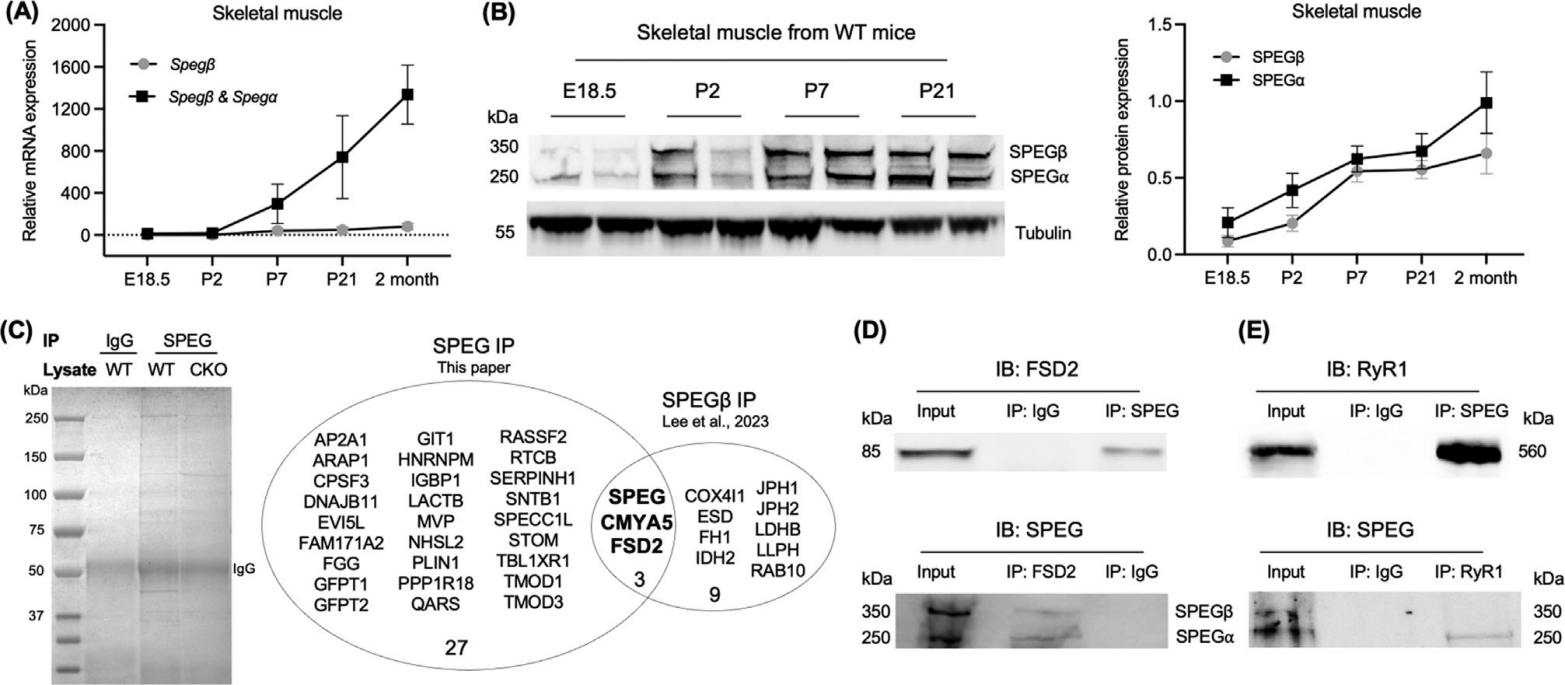
SPEG interacts with myospryn complex proteins in the skeletal muscles. (A) Quantitative real‐time PCR analysis of *Spegα* and *Spegβ* mRNA expression in the skeletal muscles of wild‐type mice and *Actb* was used as the reference gene for normalization. Relative *Spegα* and *Spegβ* mRNA expression is shown at different time points (E18.5, P2, P7, P21 and 2 months). E, embryonic; P, postnatal. (B) Immunoblot analysis for SPEGα and SPEGβ isoforms and protein quantification. Tubulin was used as a loading control. Experiments were performed using at least three different biological samples. (C) Identification of SPEG‐binding proteins in skeletal muscle by elution of SPEG–immune complexes after staining the gel with Coomassie Blue and comparing the detected proteins with the findings from Lee et al.^23^ The 30 unique SPEG‐interacting proteins were determined based on the following conditions: (1) detected only in WT muscle after SPEG immunoprecipitation (IP), (2) detected with at least four unique peptides, (3) absent in IgG IP and (4) absent in *Speg*‐CKO muscle after SPEG IP. For IP controls, skeletal muscles from WT were pre‐incubated with IgG and *Speg*‐CKO with SPEG antibodies. (D) SPEGβ and SPEGα and FSD2 co‐immunoprecipitated from skeletal muscle lysates using rabbit anti‐SPEG (that detects both SPEGβ and SPEGα) and anti‐FSD2 antibodies. (E) SPEGα and RyR1 were co‐immunoprecipitated from skeletal muscle lysates using anti‐SPEG (as above) and anti‐RyR1 antibodies.

The SPEG locus encodes for four distinct tissue‐specific isoforms: SPEGα (260 kDa) and SPEGβ (350 kDa) are highly expressed in skeletal and cardiac muscles, APEG1 in vascular tissues and BPEG in the brain and aorta.^8^ To evaluate the relative expression of SPEGα and SPEGβ over different time points, we performed quantitative real‐time PCR and immunoblot in WT mouse quadriceps muscles and found that the transcript and protein levels of SPEGα and SPEGβ gradually increased from Embryonic Day 18.5 to 2 months of age (*Figure* *1* *A*,*B*). Additionally, we compared the SPEG isoform ratio (SPEGβ/SPEGα) among the striated muscles of adult mice (including the gastrocnemius, triceps, diaphragm, soleus and heart). We found that the SPEG isoform ratios were generally consistent across different skeletal muscles, ranging from 0.82 ± 0.06 to 1.02 ± 0.19. However, the cardiac muscle exhibited a lower ratio of 0.72 ± 0.08 (*Figure* *S1*).

### SPEG interacts with myospryn complex proteins in skeletal muscle

Pooled skeletal muscles (quadriceps, gastrocnemius and triceps) from 2‐month‐old WT and *Speg*‐CKO mice were used to determine the potential binding partners of SPEG using immunoprecipitation followed by an MS assay. Following peptide search using Sequest,^16^ a list of over 350 proteins with at least four unique peptides (*Table* *S1*) was identified in the SPEG IP group using WT skeletal muscles. After excluding possible non‐specific binding partners from both IgG binding and *Speg*‐CKO IP controls, 30 SPEG‐binding candidate partners were identified (*Figure* *1* *C*).

Among these potential SPEG‐binding partners, we identified two proteins that are part of the myospryn complex (CMYA5 and FSD2). Myospryn complex is formed by CMYA5, FSD2 and ryanodine receptors and plays a critical role in the assembly of ryanodine receptor clusters and the formation of cardiac dyad architecture.^24^, ^25^ To further confirm their interaction between myospryn complex proteins (CMYA5, FSD2 and RyR1) and SPEG, we performed co‐IP experiments. Using co‐IP assays, we observed that while both SPEGβ (350 kDa) and SPEGα (250 kDa) co‐immunoprecipitated with FSD2 (*Figure* *1* *D*), only SPEGα co‐immunoprecipitated with RyR1 (*Figure* *1* *E*). The attempt to perform co‐IP using CMYA5 was unsuccessful due to the non‐specificity of the CMYA5 antibody. Interestingly, a recent study by Lee et al. also found that HA‐tagged SPEGβ interacts with both CMYA5 and FSD2 using antibodies against the HA‐tag and was identified by MS.^23^ Overall, these findings suggest that SPEG proteins are novel binding partners of the myospryn complex in skeletal muscle.

### Loss of SPEG causes myospryn complex and MTM1 abnormalities in skeletal muscles

**Figure 2:**
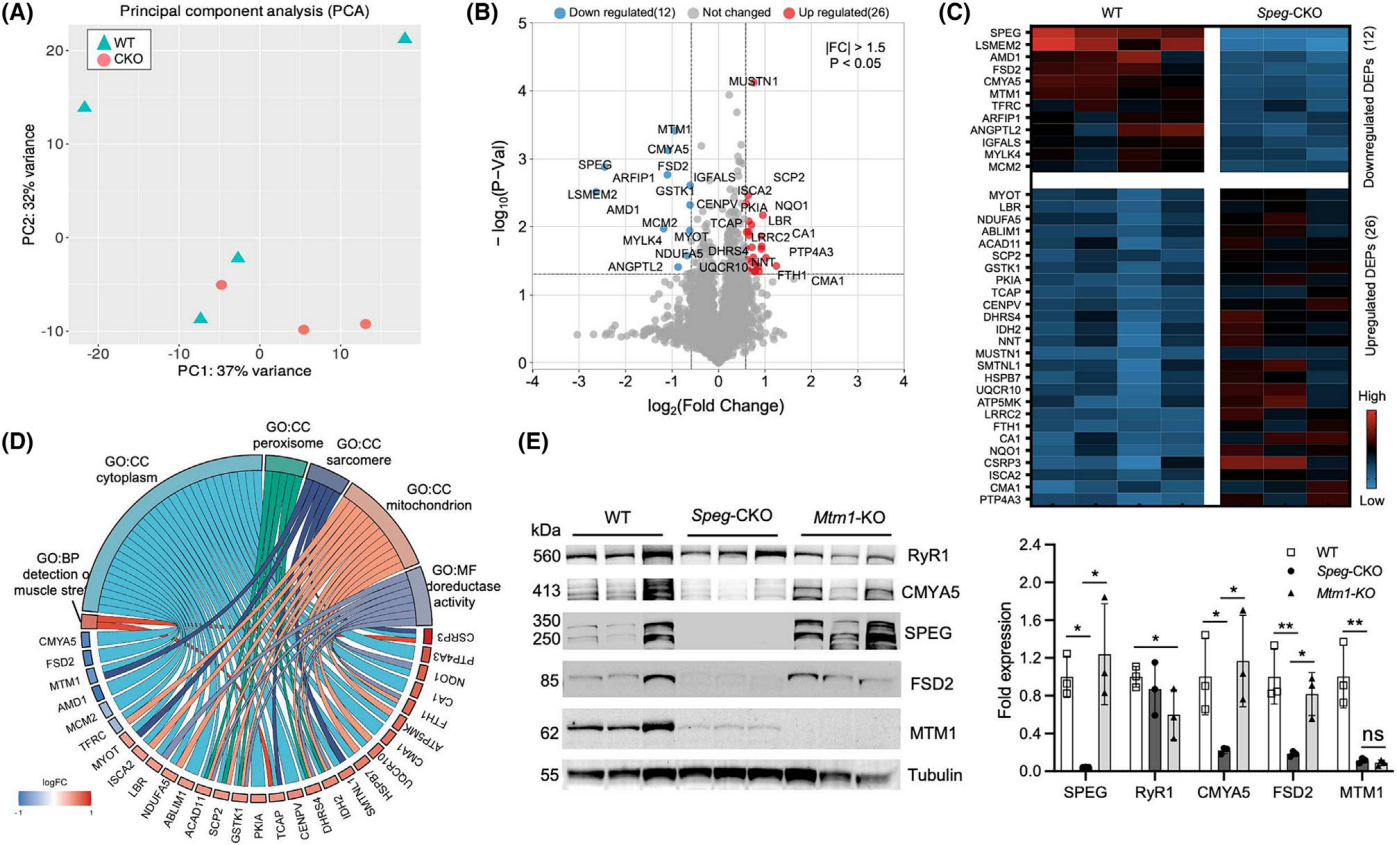
Proteomic profiling in the skeletal muscle of *Speg*‐CKO mice. (A) Principal component analysis of proteomic data. The first and second axes are represented. Coloured symbols represent genotypes for each mouse. (B) Volcano plots represent the differentially expressed proteins (DEPs). Upregulated proteins are in red, and downregulated proteins are in blue (*P* < 0.05 and a fold change beyond ±1.5). (C) Heat map of the proteins that were differentially expressed in the skeletal muscle of *Speg*‐CKO mice. (D) Chord plot displaying the enrichment analyses of DEPs for Gene Ontology analysis. (E) Immunoblot analysis and quantification of SPEG, RyR1, CMYA5, FSD2 and MTM1 relative to the expression of tubulin in the skeletal muscles of WT, *Speg*‐CKO and *Mtm1*‐KO mice (ns, not statistically significant; *P < 0.05, ^**^ *P* < 0.01, *n* = 3 per genotype; one‐way ANOVA with Tukey's post hoc test).

SPEG engages in a functional network that is critical to triad development and function by regulating the activities of triadic proteins.^9^ To assess proteomic changes associated with a lack of SPEG, quadriceps muscles from adult *Speg*‐CKO (*n* = 3) and WT mice (*n* = 4) were lysed and processed. A full list of over 3500 annotated proteins can be found in *Table* *S2*. Principal component analysis (PCA) in *Figure* *2* *A* showed that *Speg*‐CKO groups were distinct from the WT, and volcano plots (*Figure* *2* *B*) displayed the 38 DEPs (|fold change [FC]| > 1.5, *P < 0.05). Noticeably, myospryn complex protein (CMYA5 and FSD2) levels were significantly reduced in *Speg*‐CKO mice among these 38 dysregulated DEPs (*Figure* *2* *C*). GO analysis revealed enrichment for the cellular components (CCs) of the cytoplasm, mitochondria, peroxisome and sarcomere (*Figure* *2* *D*). To further validate the proteomic findings, immunoblot and quantification of select DEPs from the skeletal muscles of WT, *Speg*‐CKO and *Mtm1*‐KO mice were performed (*Figure* *2* *E*). Immunoblot findings confirmed that loss of SPEG caused a significant reduction of CMYA5 (0.22 ± 0.03, *P < 0.05), FSD2 (0.18 ± 0.03, *P < 0.01) and MTM1 levels (0.09 ± 0.03, *P < 0.01). In addition, as a comparison, skeletal muscle samples from *Cmya5*‐KO mice were obtained, which confirmed the reduction of CMYA5 in *Speg*‐CKO mice (*Figure* *S2*). In contrast, the protein levels of CMYA5, FSD2 and SPEG were similar between *Mtm1*‐KO and control mice, suggesting that this reduction in myospryn complex proteins is unique to SPEG deficiency.

### Proteomic data analysis using gene set enrichment analysis revealed alterations in mitochondrial and other proteins

**Figure 3:**
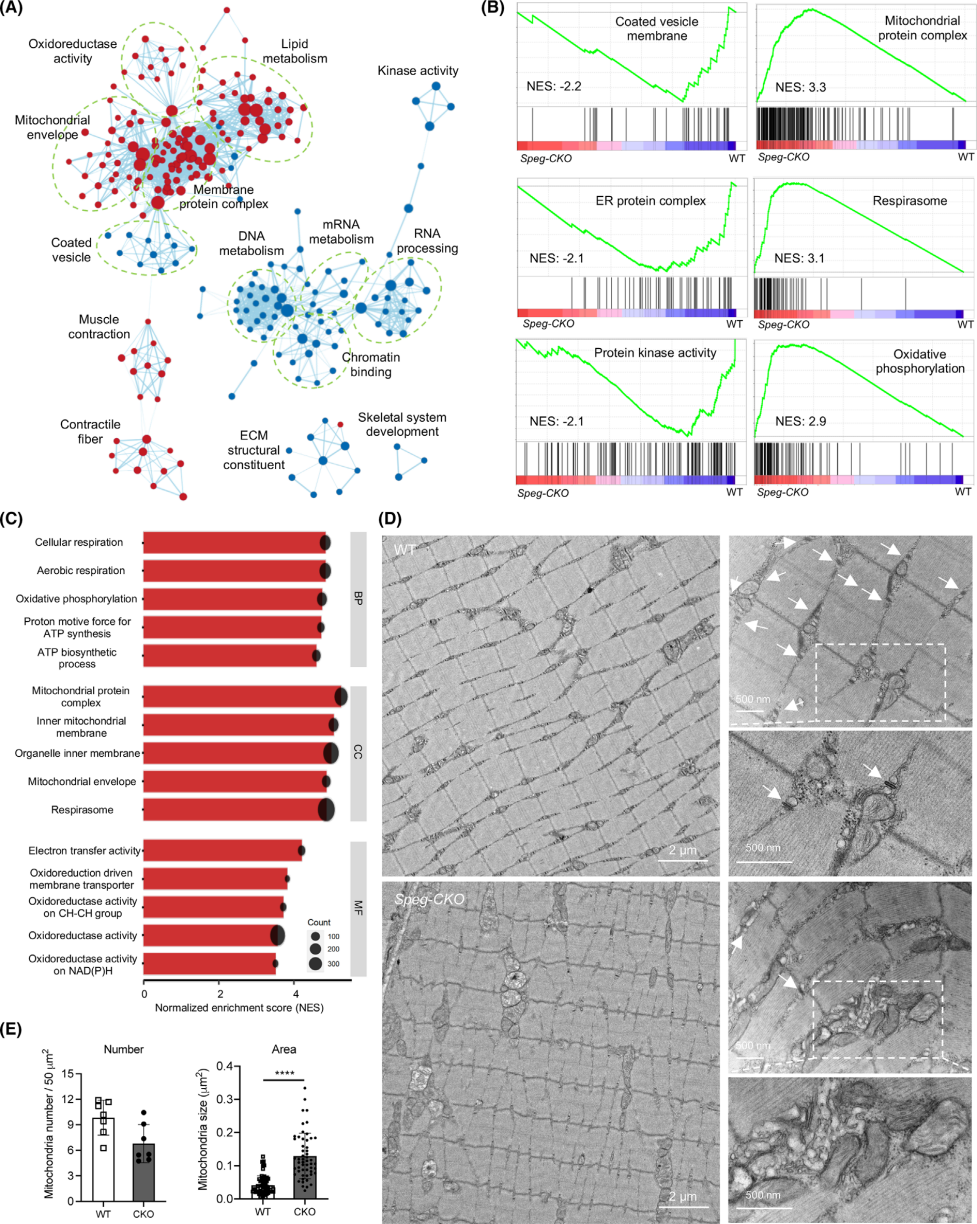
GSEA using proteomic data from the skeletal muscle of WT and *Speg*‐CKO mice and mitochondrial differences on EM. (A) The enrichment map summarizing the result of GSEA from the *Speg*‐CKO skeletal muscle proteome, presenting the downregulated (blue dots) and upregulated (red dots) protein sets in *Speg*‐CKO mice compared with WT mice. The size of a node indicates the size of each protein set, and the edge means two connected nodes share the same proteins. (B) The selected GSEA plots presenting the downregulated and upregulated protein sets in the *Speg*‐CKO mice. (C) The bar charts showing the top 5 normalized enrichment score (NES) of upregulated protein sets in the *Speg*‐CKO proteome. (D) Electron micrographs in quadriceps specimens obtained from WT and *Speg*‐CKO mice. The left panel shows an overall organization of muscle structure, and the right panel shows an enlarged view of triad (white arrows) and mitochondria (box) ultrastructure from each group. (E) Quantification of mitochondria number (left bar graph) and area (right bar graph) (^****^ *P* < 0.0001, *n* = 3 per group; unpaired two‐tailed *t* test).

To further delineate the effects of SPEG deficiency in skeletal muscles, we performed unbiased GSEA using the proteomic data from WT and *Speg*‐CKO adult quadriceps. A total of 3517 protein sets categorized by GO were visualized using the enrichment map visualization method.^26^ The red and blue nodes indicate the upregulated and downregulated protein sets, respectively. In SPEG‐deficient mice, mitochondria‐related and contractile fibre protein sets were significantly upregulated, whereas nucleic acid metabolism and ECM structural protein sets were downregulated (*Figure* *3* *A*). Select GSEA plots displaying the downregulated and upregulated protein sets in *Speg*‐CKO mice are shown in *Figure* *3* *B*. The bar charts in *Figure* *3* *C* show the top 5 normalized enrichment score (NES) of upregulated protein sets in the *Speg*‐CKO proteome, most of which are associated with mitochondrial function. We further evaluated the proteomic data for the mitochondrial contact site and cristae organizing system (MICOS) complex proteins (*Figure* *S3a*), and there were no significant changes among the detected MICOS proteins (SAM50, MICOS13, MTX1 and MTX2). Furthermore, immunoblot analysis of mitochondrial dynamics and OxPhos proteins was performed, and there was no difference between *Speg*‐CKO and WT muscles (*Figure* *S3b*).

### SPEG‐deficient muscle exhibits accumulated and swollen mitochondria

To further assess the effects of SPEG deficiency on mitochondrial morphology, sections of quadriceps muscle were examined using EM. In *Figure* *3* *D*, the *Speg*‐CKO muscle displayed structural triad anomalies (arrows) with regions of disoriented or absent triads, twisted Z lines, and abnormally accumulated and swollen mitochondria (square box). Quantitative analysis of EM findings between *Speg*‐CKO and WT muscles revealed that there was a statistically significant increase (^****^ *P* < 0.0001) in the mitochondrial area of SPEG‐deficient mice while their number remained relatively unchanged (*Figures* *3* *E* and *S3c*). Overall, these results indicate that SPEG depletion results in aberrant mitochondrial morphology in skeletal muscle.

### SPEG affects multiple phosphorylation sites on JPH2 in skeletal muscle

The SPEGα and SPEGβ proteins contain variable Ig‐like (SPEGβ: 9 Ig‐like; SPEGα: 7 Ig‐like), three fibronectin type III and two tandemly arranged serine/threonine kinase domains.^8^ The kinase function of SPEG in skeletal muscle is relatively unknown. The first kinase of SPEGα can phosphorylate JPH2 in cardiac muscle, but its phosphorylation sites are yet to be determined,^27^, ^28^ JPH2 stabilizes cardiac dyads between T‐tubule and junctional SR membranes, ensuring appropriate intracellular calcium signalling.^29^, ^30^

**Figure 4:**
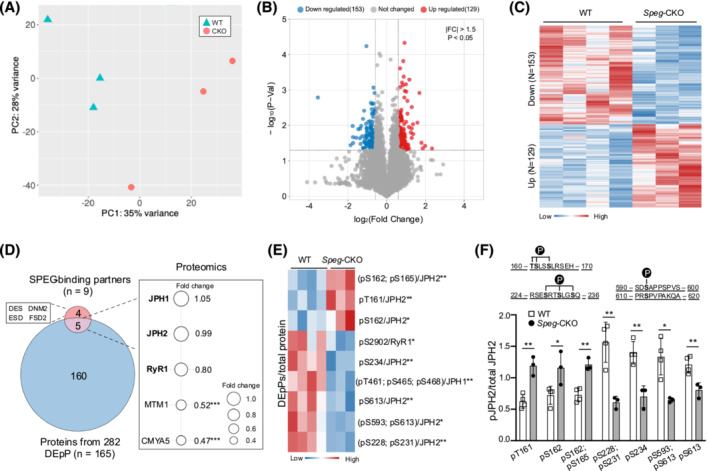
Phosphoproteomic profiling in the skeletal muscle of *Speg*‐CKO mice. (A) Principal component analysis of phosphoproteomic data. The first and second axes are represented. Coloured symbols represent genotypes for each mouse. (B) Volcano plots representing the differentially expressed phosphosites (DEpPs). Upregulated DEpPs are in red, and downregulated DEpPs are in blue (*P* < 0.05 and a fold change beyond ±1.5). (C) Heat map of the 282 DEpPs in the skeletal muscle of *Speg*‐CKO mice. (D) Left panel: Venn plot of 165 proteins from the 282 DEpPs and 9 reported SPEG‐binding partners in skeletal muscles. Right panel: overlapping protein expression identified from proteomic analysis. (E) Heat map of selected DEpPs relative to their corresponding protein level from proteomic data. (F) DEpP quantification of JPH2 at T161, S162, S165, S228, S231, S234, S593 and S613 normalized to total JPH2 protein level (*P < 0.05, ^**^ *P* < 0.01 and ^***^ *P* < 0.001; unpaired two‐tailed *t* test).

Here, we applied a phosphoproteomic approach to determine the phosphorylation substrates of SPEG in skeletal muscle. The full list of detected phosphosites can be found in *Table* *S3*. The PCA in *Figure* *4* *A* displays *Speg*‐CKO versus the WT groups, and volcano plots (*Figure* *4* *B*) illustrate the differentially expressed phosphosites (DEpPs). A total of 282 dysregulated DEpPs (129 upregulated and 153 downregulated; |FC| > 1.5, *P < 0.05) from 165 proteins were detected as shown in *Figure* *4* *C*. These DEpPs could be directly or indirectly regulated by SPEG deficiency. To identify potential DEpPs directly regulated by SPEG, we focused on the nine confirmed SPEG‐binding partners based on ours and studies from other groups. These nine proteins included RyR1, CMYA5, FSD2 (current paper), MTM1,^6^ DES,^31^ DNM2,^15^ JPH2, JPH1 and ESD.^23^ Five (JPH1, JPH2, RyR1, MTM1 and CMYA5) of those nine proteins were identified by DEpPs. Of those five proteins, only three (JPH2, RyR1 and JPH1) were further analysed as the total levels of CMYA5 and MTM1 from proteomic and WB analyses data were significantly decreased (*Figure* *4* *D*; |FC| > 1.5, ^***^ *P* < 0.001), which can explain a reduction in their site‐specific phosphorylation. *Figure* *4* *E* displays a heat map of those three selected DEpPs (that were statistically significant (*P < 0.05 and ^**^ *P* < 0.01) in relation to their corresponding protein levels. It is noteworthy that multiple DEpPs (T161, S162, S165, S228, S231, S234, S593 and S613) of JPH2 were upregulated and downregulated (*Figure* *4* *F*). JPH2 is a known binding protein and phosphorylation substrate of SPEG in cardiac muscles,^28^ and here, we identified that multiple phosphorylation sites of JPH2 were deregulated with SPEG deficiency. Additionally, other phosphorylation sites of junctional SR proteins (RyR1‐pS2902 and JPH1‐pT461, S465 and S468) were affected by the loss of SPEG.

### SPEG phosphorylates S2902 residue of RyR1

**Figure 5:**
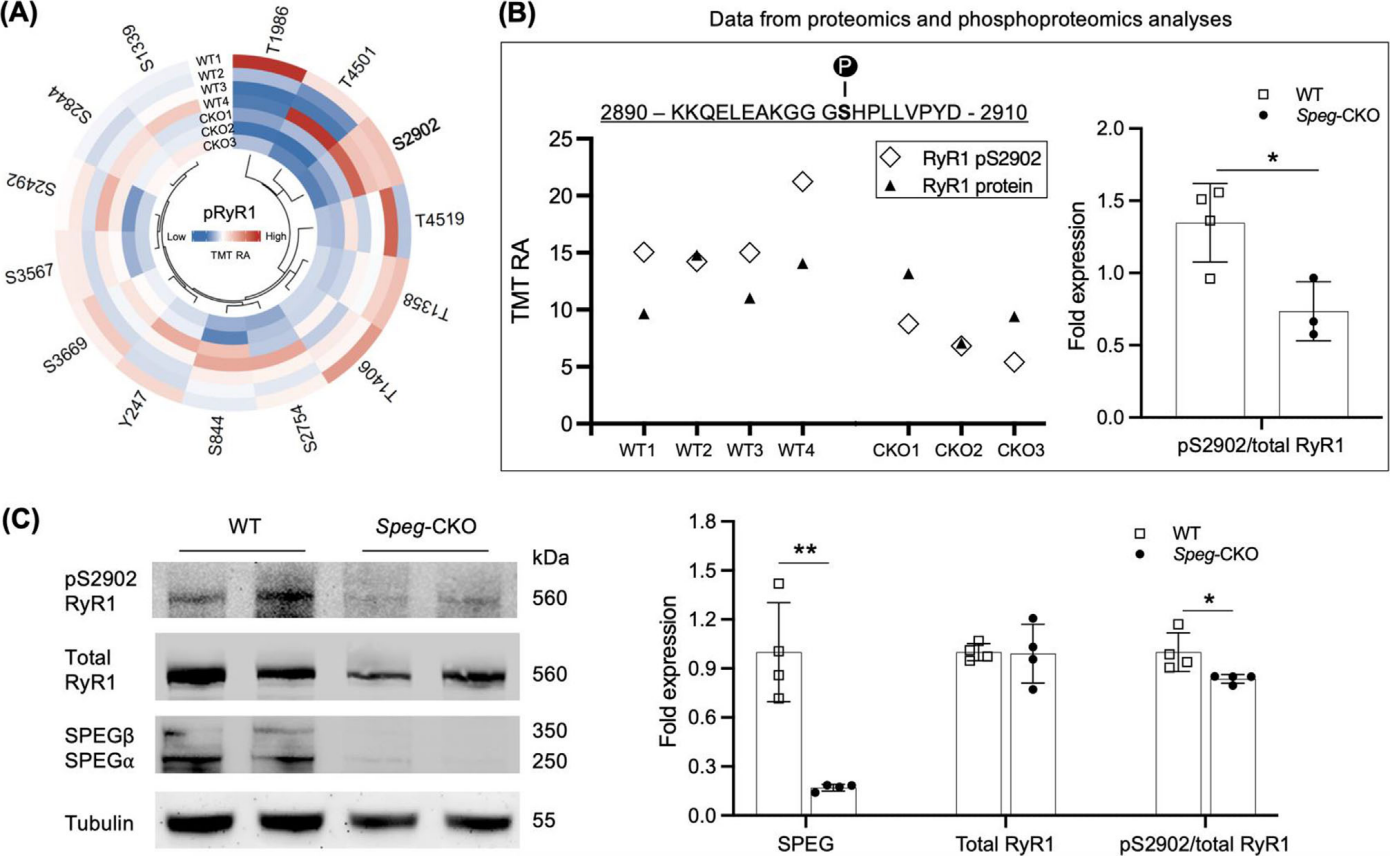
SPEG phosphorylates S2902 on RyR1 in skeletal muscle. (A) Circular heat map displaying the detected phosphosites of RyR1 by phosphoproteome. (B) RyR1 phosphoserine at 2902 (diamonds) and its associated RyR1 protein levels (triangles) detected from the proteome. Quantification of pS2902‐RyR1 levels normalized to the RyR1 protein levels (*P < 0.05; unpaired two‐tailed *t* test). (C) Immunoblot image and quantification of SPEG, pS2902‐RyR1 and total RyR1 protein levels in the skeletal muscle of WT and *Speg*‐CKO mice. A custom‐made phospho‐epitope‐specific polyclonal antibody that recognizes RyR1‐phosphorylated S2902 was generated for the immunoblot analysis (*P < 0.05 and ^**^ *P* < 0.01; *n* = 4 per group; unpaired two‐tailed *t* test).

The SPEG kinase has been reported to phosphorylate both RyR2^S2367^ (Campbell et al.^32^) and SERCA2a^T484^ (Quan et al.^27^) in cardiac muscle, which inhibits diastolic calcium release and promotes calcium reuptake into the SR, regulating calcium homeostasis between cytosol and SR. However, the role of SPEG in RyR1 phosphorylation in skeletal muscle is unknown. A previous study has reported the colocalization of SPEG and RyR1 in zebrafish myofibres, indicating a potential interaction between the two proteins.^33^ Here, we confirm their interaction using co‐IP (*Figure* *1* *E*). RyR1 is part of the myospryn complex, and it was noted to be decreased in SPEG‐deficient mice, but the reduction was not statistically significant (*Figure* *2* *E*). The phosphorylation levels of RyR1 were evaluated, and several RyR1 phosphorylation sites were detected, listed in *Figure* *5* *A*. Interestingly, one of those, pS2902‐RyR1, indicated a statistically significant difference (pS2902‐RyR1/total RyR1; *P < 0.05) confirmed by quantification analysis (*Figure* *5* *B*). To further verify this finding, a custom‐made phospho‐epitope specific polyclonal antibody that recognizes RyR1‐phosphorylated S2902 was generated and immunoblot analysis was performed (*Figure* *5* *C*). The pS2902‐RyR1 level was considerably decreased (*P < 0.05) in *Speg*‐CKO mice after correction for total RyR1 level between *Speg*‐CKO and WT mice. Thus, these data confirm that SPEG deficiency leads to decreased RyR1 phosphorylation at S2902.

### Transcriptomic analysis indicates dysregulated pathways of extracellular matrix–receptor interaction and peroxisome proliferator‐activated receptor signalling

**Figure 6:**
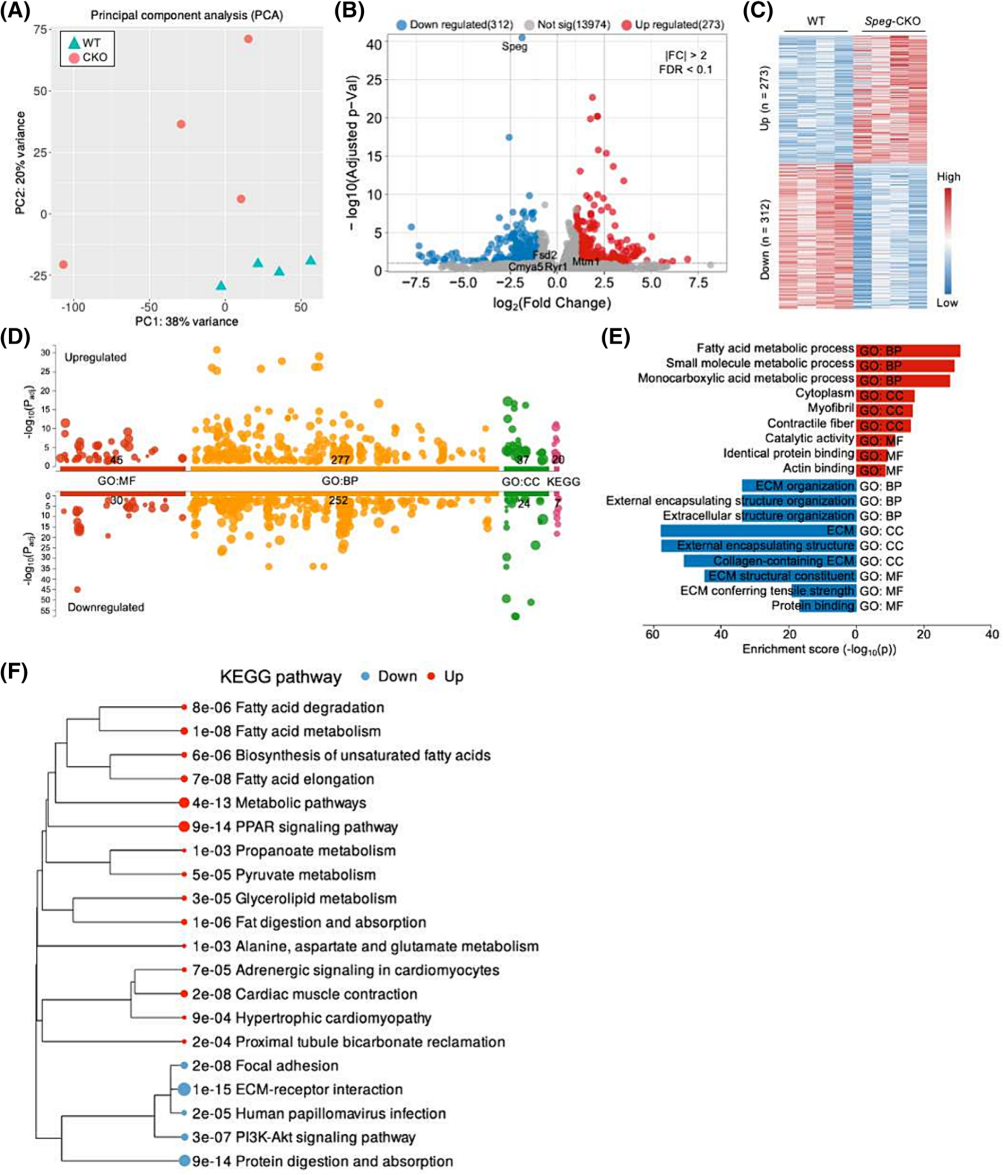
Transcriptomic profiling in the skeletal muscle of *Speg*‐CKO mice. (A) Principal component analysis of transcriptomic data. The first and second axes are represented. Coloured symbols represent genotypes for each mouse. (B) Volcano plots representing the differentially expressed genes (DEGs). Upregulated genes are in red, and downregulated genes are in blue (FDR < 0.1 and a fold change beyond ±2). (C) Heat map of the genes that were differentially expressed in the skeletal muscle of *Speg*‐CKO mice. (D) Enrichment analyses of upregulated and downregulated DEGs for Gene Ontology (GO) and KEGG pathways. The number in the *x*‐axis labels shows how many significantly enriched terms were found. The circle corresponds to term size. (E) Top 3 GO enrichment of biological process (BP), molecular function (MF) and cellular component (CC) in (D). (F) Dendrogram displaying the KEGG enrichment.

To determine molecular pathways that are dysregulated in *Speg*‐CKO mice, the quadriceps muscle transcriptomes of adult *Speg*‐CKO (*n* = 4) and WT mice (*n* = 4) were assessed. The full list of identified genes and their FCs can be found in *Table* *S4*. PCA plots revealed that *Speg*‐CKO groups separate from WT groups (*Figure* *6* *A*), and volcano plots (*Figure* *6* *B*) illustrated the DEGs. Comparing *Speg*‐CKO to WT mice, 312 DEGs were downregulated and 273 were upregulated (*Figure* *6* *C*). The downregulated and upregulated DEGs were separately enriched by GO analysis (*Figure* *6* *D*), and the top 3 GO enrichments of biological process (BP), molecular function (MF) and CC were listed in *Figure* *6* *E*. ECM and fatty acid metabolism were the most affected GO in the transcriptome of *Speg*‐CKO groups. Additionally, DEGs were also used for KEGG pathway analysis (*Figure* *6* *F*), and pathways of ECM–receptor interaction and peroxisome proliferator‐activated receptor (PPAR) signalling were the most dysregulated in *Speg*‐CKO groups. While genes linked to the ECM–receptor interaction pathway were generally downregulated (*Figure* *S4*), those linked to the PPAR signalling pathway ones were predominantly upregulated (*Figure* *S5*). Taken together, these findings suggest that SPEG deficiency affects the transcription of ECM and PPAR pathway‐related genes in skeletal muscles.

### Poor correlation between transcriptomic and proteomic data

The Pearson correlation analysis of transcriptomic and proteomic data revealed a weak correlation (0.22; *Figure* *S6*) between detected transcripts and proteins, likely because of low expression and inconsistency between RNA and protein activity due to post‐translational and other modifications.^34^ For example, the transcriptomic data suggested that SPEG deficiency did not affect the transcript levels of the myospryn complex (CMYA5, FSD2 and RyR1) in contrast to the marked reduction of protein levels. This was further confirmed by quantitative real‐time PCR (qRT‐PCR) (*Figure* *S7*) and immunoblot analysis.

## Discussion

We applied multi‐omics approaches and follow‐up experiments to elucidate the fundamental role of SPEG in skeletal muscle function, which is critical for determining the molecular basis of SPEG‐related diseases and exploring novel therapies.

We have identified that SPEG is a novel binding partner of the myospryn complex, which includes CMYA5, FSD2 and RyR1 proteins. Recent studies have revealed that CMYA5 and FSD2 are concentrated at the dyad in cardiac muscle and the triad in skeletal muscle, respectively, and they co‐localize with ryanodine receptors at the junctional SR.^24^ However, their role in skeletal muscle is poorly understood. CMYA5 is a large tripartite motif‐containing protein (~413 kDa), while FSD2 (fibronectin type III and SPRY domain‐containing protein 2) is a protein (~85 kDa) closely related to the C‐terminus of CMYA5. CMYA5 was previously reported to interact with various muscle proteins, including α‐actinin, desmin, dysbindin, the RIIα regulatory subunit of PKA, dystrophin, calcineurin, titin and calpain‐3, with all these interactors binding to overlapping regions in the C‐terminal tripartite motif of CMYA5.^24^ Recently, Lee et al. identified CMYA5, FSD2, JPH1, JPH2 and ESD as SPEGβ binding partners using HA‐tag.^23^ In this study, we also found that SPEG not only interacts with myospryn complex, but in addition, its deficiency is associated with a significant reduction in levels of myospryn complex proteins with unchanged transcript levels, indicating that SPEG plays a critical role in the regulation of myospryn complex in skeletal muscle.

Prior research from us and others has elucidated the critical role of SPEG in regulating calcium homeostasis in striated muscles. In cardiac muscle, SPEG can phosphorylate both RyR2^S2367^ (Campbell et al.^32^) and SERCA2a^T484^ (Quan et al.^27^), which could inhibit diastolic calcium release and promote calcium reuptake into the SR. In skeletal muscle, we have previously shown that loss of SPEG affected the amplitude and the kinetics of RyR‐mediated SR calcium release in 3‐month‐old *Speg*‐CKO mice.^9^ Here, we confirmed the interaction between SPEG and RyR1 and further identified that RyR1‐S2902 is a novel kinase substrate of SPEG. Additionally, SPEG loss alters the phosphorylation level of JPH2 at multiple sites, which may be involved in calcium release from the RyR complex.^28^, ^35^ We hypothesize that loss of RyR1 and JPH2 phosphorylation due to SPEG deficiency decreases RyR1‐mediated SR calcium release in the junctional membrane complex of skeletal muscle, which may trigger the disruption of T‐tubules and JPH2 cleavage.

We have previously demonstrated that SPEGβ interacts with desmin and DNM2,^15^, ^31^ and SPEGα interacts with MTM1 using co‐IP.^6^ In our interactomic experiments, MTM1 was not detected in WT muscles after SPEG IP, likely due to low‐affinity interactions during the washes and/or short half‐time. Meanwhile, DES, DNM2 and RyR1, three previously reported interactors, were present in the *Speg*‐CKO mice, albeit in lower amounts compared to WT. We suspect it may be due to the residual expression of SPEG protein in *Speg*‐CKO mice and/or the non‐specificity of the SPEG antibody. Furthermore, due to the large amounts of protein needed, IP‐MS was performed only once, which may result in skewed reporting and/or missing of potential interacting proteins. To overcome this, we performed co‐IP assays to further verify interactions for proteins of our interest. Here, we identified that antibody against FSD2 pulled down both SPEGα and SPEGβ, while RyR1 IP only pulled down SPEGα. There is no effective antibody available against CMYA5 to confirm its interaction with SPEG using co‐IP.

Patients with mutations affecting both SPEG isoforms are associated with a more severe clinical phenotype, while those with mutations affecting only SPEGβ are associated with a milder phenotype and often without cardiac involvement.^7^, ^36^ In the Lee et al. study, they created a new mouse model with SPEGβ deficiency (HA‐*Speg* mice) and found that HA‐*Speg* mice display mild muscle weakness with no cardiac involvement.^23^ Indeed, SPEGα is essential for both skeletal and cardiac function and may partially compensate for SPEGβ deficiency.^36^ Here, we list the unique interacting partners of SPEGα and SPEGβ in skeletal muscle (*Table* *1*). Based on the analysis of interacting partners and patient phenotypes, we suspect that SPEGα and SPEGβ may have overlapping yet unique functions.

**Table 1:** SPEG and its interacting proteins in skeletal muscle

SPEG isoform	Region of SPEG	Interacting proteins	Protein location	Function in triad/dyad	Reference
SPEGα	Unknown	RyR1	SR terminal cisternae	Calcium channel that mediates the release of Ca^2+^ from the SR into the cytosol	This study
	Ig‐like 9 and part of FnIII‐2 domain (aa: 2530–2674)	MTM1 Phosphatase and coiled‐coil domains (aa: 155–603)	SR terminal cisternae	T‐tubule biogenesis and triad formation	Agrawal et al. study^6^
SPEGβ	Ig‐like 9 and FnIII‐2 domain (aa: 2200–2960)	Desmin Rod domain (aa: 179–228)	Z line	Maintaining sarcomere structure, interconnecting Z‐discs and forming myofibrils, linking them to the sarcolemmal cytoskeleton, nucleus and mitochondria	Luo et al. study^31^
	Unknown	DNM2	Z line	T‐tubule formation	Li et al. study^15^
	Unknown	CMYA5^a^	Both Z line and SR terminal cisternae	Assembly of ryanodine receptor clusters in striated muscle	Lee et al. study^23^
	Unknown	FSD2	Both Z line and SR terminal cisternae	Work with CMYA5 as myospryn complex	
	Unknown	ESD	Triad	Serine hydrolase	
Both isoforms	Unknown	CMYA5^a^	Both Z line and SR terminal cisternae	Assembly of ryanodine receptor clusters in striated muscle	This study
	Unknown	FSD2	Both Z line and SR terminal cisternae	Work with CMYA5 as myospryn complex	

Abbreviations: aa, amino acid; SR, sarcoplasmic reticulum.

^a^ Additional co‐immunoprecipitation study will be required to confirm their interaction.

The mitochondrial abnormalities seen in SPEG‐deficient muscles may be secondary to defects in triad and calcium homeostasis. In muscle cells, calcium is released from the SR, and defects in the triad can lead to abnormal calcium signalling that affects mitochondrial function. Calcium regulates key enzymes involved in mitochondrial oxidative phosphorylation, the process by which mitochondria generate ATP, and can also stimulate mitochondrial biogenesis and mitochondrial fusion.^37^ Recent findings show that interactions among SPEG, MTM1, DNM2 and BIN1 are essential for triad development and function.^15^, ^38^, ^39^ Triad and mitochondrial abnormalities have been seen with *Mtm1*‐KO, *Bin1*‐KO and *Dnm2*‐KI mice (*Table* *S5*), and similarly, defective or absent triads and abnormally accumulated swollen mitochondria were detected in *Speg*‐CKO muscle.^40^ Here, GSEA analysis of DEPs reveals upregulation of mitochondrial oxidoreductase, membrane protein complex and lipid metabolism in *Speg*‐CKO mice. Meanwhile, GO analysis of DEGs indicates significantly upregulated PPAR signalling pathway, fatty acid elongation and metabolism. PPARs are a class of nuclear receptors that play crucial roles in development and energy metabolism, and they are master regulators of glucose and lipid homeostasis as well as modulators of mitochondrial function.^41^ Overall, the upregulation of the PPAR signalling pathway and mitochondrial functions likely acts to compensate for the triadic defects and calcium mishandling in the skeletal muscle of *Speg*‐CKO mice.

In summary, we show that SPEG interacts with myospryn complex proteins in the skeletal muscle, and its deficiency results in myospryn complex abnormalities and a decreased RyR1 phosphorylation level at S2902. Additionally, we identify that SPEG phosphorylates multiple phosphorylation sites of JPH2, suggesting the critical phosphorylation function of the kinase domains of SPEG. We also determined that SPEGα and SPEGβ have different interacting partners, which suggests their unique functions. Furthermore, the mitochondrial defects seen in *Speg*‐CKO skeletal muscle may be a consequence of abnormal calcium signalling. This study demonstrates the advantages of using multi‐omics techniques to comprehensively analyse the pathological and molecular anomalies of rare diseases, setting the groundwork for the development of novel and precise therapeutics for patients carrying *SPEG* mutations.

## Conflict of interest statement

The authors declare that they have no conflicts of interest.

## Supporting information


**Figure S1.** Immunoblot analysis of SPEG isoform ratio (SPEGβ/ SPEGα) among mouse striated muscles. Tubulin is used as a loading control. Quad: quadriceps; Gas: gastrocnemius.
**Figure S2.** Immunoblot analysis of CMYA5 relative to the expression of tubulin in quadriceps muscle of WT, *Speg*‐CKO, and *Cmya5*‐KO mice.
**Figure S3.** No statistical difference in mitochondria associated proteins between *Speg*‐CKO and WT muscles. (a) Heat map of mitochondrial contact site and cristae organizing system complex proteins detected from proteome data. (b) Immunoblot analysis and quantification of mitochondrial OxPhos (CI subunit NDUFB8, CII subunit SDHB, CIII‐Core protein 2 (UQCRC2), CIV subunit I (MTCO1), and CV alpha subunit (ATP5A)) and dynamics proteins (MFF: mitochondrial fission factor) relative to the expression of tubulin in skeletal muscles of WT and *Speg*‐CKO mice (*n* ≥ 4 per genotype). (c) Electron micrographs in quadriceps from WT and *Speg*‐CKO mice.
**Figure S4.** Dysregulated transcripts detected in the pathways of ECM‐receptor interaction and heat map of these genes that were detected in the ECM‐receptor interaction by transcriptome analysis.
**Figure S5.** Dysregulated transcripts detected in the pathways of peroxisome proliferator‐activated receptors signaling and heat map of these genes that were detected in the PPAR signaling pathways by transcriptome analysis.
**Figure S6.** No strong correlation was found in the Pearson correlation analysis of proteomic and transcriptome data. Pearson correlation between mRNA and protein levels measured by RNASeq and mass spectrometry using skeletal muscles of WT and *Speg*‐CKO mice.
**Figure S7.** Real‐time quantitative PCR (qRT‐PCR) analysis for genes of interest using mRNA extracted from mouse skeletal muscle. *Actb* as the reference gene for normalization (*P < 0.05, *n* = 4 per group; unpaired 2‐tailed t test). qRT‐PCR primers were listed in Table S6.

**Table S1.** Detected proteins in the SPEG IP muscle lysate of WT and Speg‐CKO skeletal muscle using mass spectrometry.

**Table S2.** List of detected proteins from proteomics analysis.

**Table S3.** The list of detected phosphosites from phosphoproteomic analysis.

**Table S4.** The list of detected genes from transcriptomic analysis.

**Table S5.** Mitochondrial findings in skeletal muscles from Speg‐CKO, Mtm1‐KO, Dnm2‐KI, and Bin1‐KO mice.

**Table S6.** Primer sequences for quantitative PCR amplification.
