# Supplementary material for: Integrated multi‐omics approach reveals the role of striated muscle preferentially expressed protein kinase in skeletal muscle including its relationship with myospryn complex
Source: J Cachexia Sarcopenia Muscle. 2024 May 9;15(3):1003–15. doi: 10.1002/jcsm.13470 (PMC11154751; doi:10.1002/jcsm.13470)
Supplement: Supplementary file 1 — Figure S1. Immunoblot analysis of SPEG isoform ratio (SPEGβ/ SPEGα) among mouse striated muscles. Tubulin is used as a loading control. Quad: quadriceps; Gas: gastrocnemius. Figure S2. Immunoblot analysis of CMYA5 relative to the expression of tubulin in quadriceps muscle of WT, Speg‐CKO, and Cmya5‐KO mice. Figure S3. No statistical difference in mitochondria associated proteins between Speg‐CKO and WT muscles. (a) Heat map of mitochondrial contact site and cristae organizing system complex proteins detected from proteome data. (b) Immunoblot analysis and quantification of mitochondrial OxPhos (CI subunit NDUFB8, CII subunit SDHB, CIII‐Core protein 2 (UQCRC2), CIV subunit I (MTCO1), and CV alpha subunit (ATP5A)) and dynamics proteins (MFF: mitochondrial fission factor) relative to the expression of tubulin in skeletal muscles of WT and Speg‐CKO mice (n ≥ 4 per genotype). (c) Electron micrographs in quadriceps from WT and Speg‐CKO mice. Figure S4. Dysregulated transcripts detected in the pathways of ECM‐receptor interaction and heat map of these genes that were detected in the ECM‐receptor interaction by transcriptome analysis. Figure S5. Dysregulated transcripts detected in the pathways of peroxisome proliferator‐activated receptors signaling and heat map of these genes that were detected in the PPAR signaling pathways by transcriptome analysis. Figure S6. No strong correlation was found in the Pearson correlation analysis of proteomic and transcriptome data. Pearson correlation between mRNA and protein levels measured by RNASeq and mass spectrometry using skeletal muscles of WT and Speg‐CKO mice. Figure S7. Real‐time quantitative PCR (qRT‐PCR) analysis for genes of interest using mRNA extracted from mouse skeletal muscle. Actb as the reference gene for normalization (*P < 0.05, n = 4 per group; unpaired 2‐tailed t test). qRT‐PCR primers were listed in Table S6. [file JCSM-15-1003-s001.docx]

**Integrated multi-omics approach reveals the role of SPEG in skeletal muscle including its relationship with myospryn complex**

Qifei Li^1,2,3,^, Jasmine Lin^2,3^, Shiyu Luo^1,2,3^, Klaus Schmitz-Abe^1,2,3^, Rohan Agrawal^1,2,3^, Melissa Meng^2,3^, Behzad Moghadaszadeh^2,3^, Alan H. Beggs^2,3^, Xiaoli Liu^4,5^, Mark A. Perrella^4,5^, Pankaj B. Agrawal^1,2,3*^

^1^Division of Neonatology, Department of Pediatrics, University of Miami Miller School of Medicine and Holtz Children's Hospital, Jackson Health System, Miami, FL, USA

^2^Division of Genetics and Genomics, ^3^The Manton Center for Orphan Disease Research, Boston Children’s Hospital, Harvard Medical School; Boston, Massachusetts 02115, USA.

^4^Division of Pulmonary and Critical Care Medicine, ^5^Department of Pediatric Newborn Medicine, Brigham and Women’s Hospital, Harvard Medical School; Boston, Massachusetts 02115, USA.

*To whom correspondence should be addressed at: Pankaj B. Agrawal, MD, ^4^Division of Neonatology, Department of Pediatrics, University of Miami Miller School of Medicine, Miami, FL, USA. Tel: 305-585-6408; Email: pagrawal@miami.edu

**Supplementary Materials**

**Methods**

*Immunoblot analysis.* Skeletal muscles from WT, *Speg*-CKO, and *Mtm1*-KO mice were dissected, snap frozen in isopentane, and stored at −80°C until analysis. Protein isolation and western blot procedures were performed as described previously [1]. Immunofluorescent western blot was performed. Proteins were probed with primary antibodies against rabbit anti-SPEG (12472-T16, 1:1000 dilution, SinoBiological, Beijing, China), mouse anti-RyR1 (sc-376507, 1:800 dilution, Santa Cruz Biotechnology), rabbit anti-CMYA5 (1:800 dilution, from Dr. Francisco J. Naya, Boston University), rabbit anti-MTM1 (PI168, 1:800 dilution, from IGBMC), mouse anti-FSD2 (sc-393072, 1:1000 dilution, Santa Cruz Biotechnology), and custom rabbit-anti-RyR1-pS2902 (YenZym, South San Francisco, CA; 1:800). IRDye 800CW Donkey anti-Rabbit IgG Secondary antibody (926-32213, 1:5000, LI-COR), IRDye 680RD Donkey anti-Mouse IgG Secondary antibody (926-68072, 1:5000, LI-COR), and anti-tubulin Rhodamine antibody (AbD22584, 1:5000, Bio-Rad Laboratories, Hercules, CA, USA) were used for immunofluorescence detection. Quantification of protein levels normalized to tubulin was performed using ImageJ software.

*Quantitative PCR with reverse transcription (RT–qPCR).* Total RNA was isolated from skeletal muscles of WT and *Speg*-CKO mice using the mirVana miRNA Isolation Kit (AM1561, Thermofisher) according to manufacturer’s protocol. One µg of total RNA was used to generate complementary DNA (cDNA) using the SuperScript™ IV First-Strand Synthesis System and random hexamers (cat# 18091050, ThermoFisher Scientific). qRT- PCR was performed using PowerUp SYBR Green Master mix (A25742, Thermofisher) in a QuantStudio™ 3 Real-Time PCR System. Real-time quantitative PCR (qRT-PCR) primers were listed in **Table S6**.

*Identification of the interactome*. Immunoprecipitation of SPEG was performed on pooled skeletal muscles from at least 3 WT mice for each experiment. A rabbit anti-SPEG antibody (12472-T16, 1:50 dilution, SinoBiological, Beijing, China) was used for immunoprecipitation. Samples were concentrated and analyzed by SDS–PAGE and stained with the Coomassie blue (1610786, Bio-Rad Laboratories, Hercules, CA, USA). The IgG control and the *Speg*-CKO lysate incubated with SPEG antibody were used as controls. Excised gel bands were cut into approximately 1 mm^3^ pieces. Gel pieces were then subjected to a modified in-gel trypsin digestion procedure [2]. Gel pieces were washed and dehydrated with acetonitrile for 10 min. followed by removal of acetonitrile. Pieces were then completely dried in a speed-vac. Rehydration of the gel pieces was with 50 mM ammonium bicarbonate solution containing 12.5 ng/µl modified sequencing-grade trypsin (Promega, Madison, WI) at 4ºC. After 45 min., the excess trypsin solution was removed and replaced with 50 mM ammonium bicarbonate solution to just cover the gel pieces. Samples were then placed in a 37ºC room overnight. Peptides were later extracted by removing the ammonium bicarbonate solution, followed by one wash with a solution containing 50% acetonitrile and 1% formic acid. The extracts were then dried in a speed-vac (~1 hour).

The samples were reconstituted in 5 - 10 µl of HPLC solvent A (2.5% acetonitrile, 0.1% formic acid). A nano-scale reverse-phase HPLC capillary column was created by packing 2.6 µm C18 spherical silica beads into a fused silica capillary (100 µm inner diameter x ~30 cm length) with a flame-drawn tip [3]. After equilibrating the column each sample was loaded via a Famos auto sampler (LC Packings, San Francisco CA) onto the column. A gradient was formed and peptides were eluted with increasing concentrations of solvent B (97.5% acetonitrile, 0.1% formic acid). As peptides eluted, they were subjected to electrospray ionization and then entered an LTQ Orbitrap Velos Pro ion-trap mass spectrometer (Thermo Fisher Scientific, Waltham, MA). Peptides were detected, isolated, and fragmented to produce a tandem mass spectrum of specific fragment ions for each peptide. Peptide sequences (and hence protein identity) were determined by matching protein databases with the acquired fragmentation pattern by the software program, Sequest (Thermo Fisher Scientific, Waltham, MA) [4]. All databases include a reversed version of all the sequences and the data was filtered to between a one and two percent peptide false discovery rate.

*Phosphoproteome profiling.* Skeletal muscle samples of WT (n = 4) and *Speg*-CKO (n = 3) mice were lysed and processed as described in the SPEED protocol [5]. Following digestion with Lys-C and trypsin, peptides were desalted by using 100mg SepPak columns. The elutions were dried via vacuum centrifugation and the phosphopeptides were enriched with the High-Select Fe^3+^-NTA Phosphopeptide Enrichment Kit according to manufacturer’s specifications using approximately 1 mg protein digest per enrichment column. The elutions were dried via vacuum centrifugation, while the flow-throughs were saved for subsequent whole proteome analysis.

TMT labeling. In general, we estimate that at most 10 ug of phosphopeptides were enriched from 1 mg of total peptide. We added 20 ug of TMTpro reagent (Thermo-Fisher; Lot #:WL338745) to the peptides (~10 µg) along with acetonitrile to again achieve a final acetonitrile concentration of approximately 30% (v/v) in a total volume of 50 µL. Following incubation at room temperature for 1 h, the reaction was quenched with hydroxylamine to a final concentration of 0.3% (v/v). The sample was vacuum centrifuged to near dryness and subjected to C18 solid-phase extraction (SPE, Sep-Pak).

Off-line basic pH reversed-phase (BPRP) fractionation. We fractionated the peptide samples using BPRP HPLC. We used an Agilent 1200 pump equipped with a degasser and a UV detector. Peptides were subjected to a 50-min linear gradient from 5% to 35% acetonitrile in 10 mM ammonium bicarbonate pH 8 at a flow rate of 0.6 mL/min over an Agilent 300Extend C18 column (3.5 μm particles, 4.6 mm ID and 250 mm in length). The peptide mixture was fractionated into a total of 96 fractions, which were consolidated into 24 super-fractions (in a checkerboard-like pattern). Samples were subsequently acidified with 1% formic acid and vacuum centrifuged to near dryness. Each consolidated fraction was desalted by StageTip, and reconstituted in 5% acetonitrile, 5% formic acid for LC-MS/MS processing.

Mass spectrometric data were collected on an Orbitrap Eclipse mass spectrometer coupled to a Proxeon NanoLC-1200 UHPLC (ThermoFisher Scientific). The 100 µm capillary column was packed in-house with 35 cm of Accucore 150 resin (2.6 μm, 150Å; ThermoFisher Scientific). Data were acquired for 120 min per fraction. The scan sequence began with an MS1 spectrum: Orbitrap analysis, resolution 120,000, 400−1500 Th, automatic gain control (AGC) target 400K, maximum injection time 50 ms. MS2 analysis, which occurred in the OrbiTrap, consisted of higher-energy collision dissociation (HCD), AGC 150K, NCE (normalized collision energy) 36, isolation window 0.5 Th, maximum injection time set to 250 and TopSpeed set at 1.5 sec. Each of the samples were shot twice using two sets of compensation voltages. For FAIMS, the dispersion voltage (DV) was held constant at 5000V, the compensation voltages (CVs) were set at -35V, -55V, and -75V for the first shot and -45 and -65V for the second shot. In total each of the 12 fractions were run twice, for a total of 24 runs.

Database searching included all entries from the mouse UniProt Database (downloaded: 2021). The database was concatenated with one composed of all protein sequences for that database in the reversed order [6]. Raw files were converted to mzXML, and monoisotopic peaks were re-assigned using Monocle [7]. Searches were performed with Comet [8] using a 50-ppm precursor ion tolerance for total protein level profiling. The product ion tolerance was set to 0.02 Da. TMTpro labels on lysine residues and peptide N-termini (+304.207 Da), as well as carbamidomethylation of cysteine residues (+57.021 Da) were set as static modifications, while oxidation of methionine residues (+15.995 Da), phosphorylation (+79.966), and deamidation (+0.984) were set as variable modifications. Peptide-spectrum matches (PSMs) were adjusted to a 1% false discovery rate (FDR) using a linear discriminant after which proteins were assembled further to a final protein-level FDR of 1% analysis [9]. AScore was used to determine site localization [10], with a score of 13 denoting 95% confidence for a specified phosphorylation site. Phosphorylation sites were quantified by summing reporter ion counts across all matching PSMs. More specifically, reporter ion intensities were adjusted to correct for the isotopic impurities of the different TMTpro reagents according to manufacturer specifications. Peptides were filtered to include only those with a summed signal-to-noise (SN) ≥ 100 across all TMT channels. An extra filter of an isolation specificity (“isolation purity”) of at least 0.5 in the MS1 isolation window was applied for the phosphorylated peptide analysis. The signal-to-noise (S/N) measurements of peptides were globally normalized using the protein normalization factors mentioned below to account for equal protein loading. Cutoff values for differentially expressed phosphorylation peptide determinations were as follows: p value <0.05 and absolute value of log2FC >1.5.

*Whole proteome profiling.* Flow-throughs from the phospho-enrichment described above, 50 µg per replicate, were used for the whole proteome work for each sample. 120 µg of TMTpro reagents (Thermo-Fisher; Lot #:WL338745) were added to the peptides (50 µg) along with acetonitrile to achieve a final acetonitrile concentration of approximately 30% (v/v) in a total volume of 100 µL. Following incubation at room temperature for 1 h, the reaction was quenched with hydroxylamine to a final concentration of 0.3% (v/v). The sample was vacuum centrifuged to near dryness and subjected to C18 solid-phase extraction (SPE, Sep-Pak). Off-line basic pH reversed-phase (BPRP) fractionation was the same as phosphoproteome profiling.

Mass spectrometric data were collected on an Orbitrap Fusion Lumos mass spectrometer coupled to a Proxeon NanoLC-1200 UHPLC (ThermoFisher Scientific). A 100 µm capillary column was packed in-house with 35 cm of Accucore 150 resin (2.6 μm, 150Å; ThermoFisher Scientific). Data were acquired for 90 min per fraction. The scan sequence began with an MS1 spectrum: Orbitrap analysis, resolution 60,000, 400−1600 Th, automatic gain control (AGC) target 400K, maximum injection time 50 ms. MS2 analysis consisted of collision-induced dissociation (CID), quadrupole ion trap analysis, AGC 10K, NCE (normalized collision energy) 35, q-value 0.25, isolation window 0.6 Th, maximum injection time set to 35 and TopSpeed set at 1.25 sec. An on-line real-time search algorithm (Orbiter) was used to trigger MS3 scans for quantification [11]. For the MS3 scan (performed in the OrbiTrap), we used higher-energy collision dissociation (HCD) with NCE 55%, AGC 200K, maximum injection time 200 ms, resolution 50,000 at 400 Th, isolation window 1.2. The close out was set at two peptides per protein per fraction [11]. For High-field Asymmetric-waveform Ion Mobility spectrometry (FAIMS), the dispersion voltage (DV) was held constant at 5000V, the compensation voltages (CVs) were set at -40V, -60V, and -80V [12]. In total 24 fractions were analyzed for each multiplexed experiment.

Searches were performed as described above but with product ion tolerance was set to 0.9 Da (as the MS2 scans are low-resolution) and without phosphorylation or deamidation variable modifications. Proteins were quantified by summing reporter ion counts across all matching PSMs. More specifically, reporter ion intensities were adjusted to correct for the isotopic impurities of the different TMTpro reagents according to manufacturer specifications. Peptides were filtered to include only those with a summed signal-to-noise (SN) ≥ 100 across all TMT channels. The signal-to-noise (S/N) measurements of peptides assigned to each protein were summed (for a given protein). These values were normalized so that the sum of the signal for all proteins in each channel was equivalent thereby accounting for equal protein loading. The resulting normalization factors will be used to normalize the phosphorylation sites as discussed above to account for equal protein loading. Finally, each protein abundance measurement was scaled, such that the summed signal-to-noise for that protein across all channels equals 100, thereby generating a relative abundance (RA) measurement. Cutoff values for differentially expressed protein (DEPs) determinations were as follows: p value <0.05 and absolute value of fold change >1.5.

*Transcriptome profiling*. Skeletal muscles from quadriceps femoris were isolated from WT and *Speg*-CKO mice. A total of 8 mRNA samples (n = 4 for per group) was isolated and was then converted into double-stranded DNA (dsDNA). Procedures including the sample quality control (QC), the library preparation, and sequencing for all the samples were performed by Novogene (Sacramento, CA, USA). RNA-seq raw data were aligned using “align” (Rsubread, V2.0.1) and the latest UCSC mouse annotation (GRCm38/mm10). The raw data were trimmed before aligned using Trimmomatic (V0.39) for QC. Gene-level read counts were quantified using “featureCounts” (Rsubread, V2.0.1). To identify differentially expressed genes, DESeq2 (version 1.26.0) was used with default parameters in Bioconductor packages. All software/packages were run using their default parameters. Count tables were normalized to TPM (Transcripts per Million) for visualizations and QC. Sample clustering and standard path analyses (GO and KEGG) were performed using a custom-made pipeline (VExP -RNseq). Transcripts were called as differentially expressed when the adjusted p values were below 0.1 and fold-changes were over ±2.

**
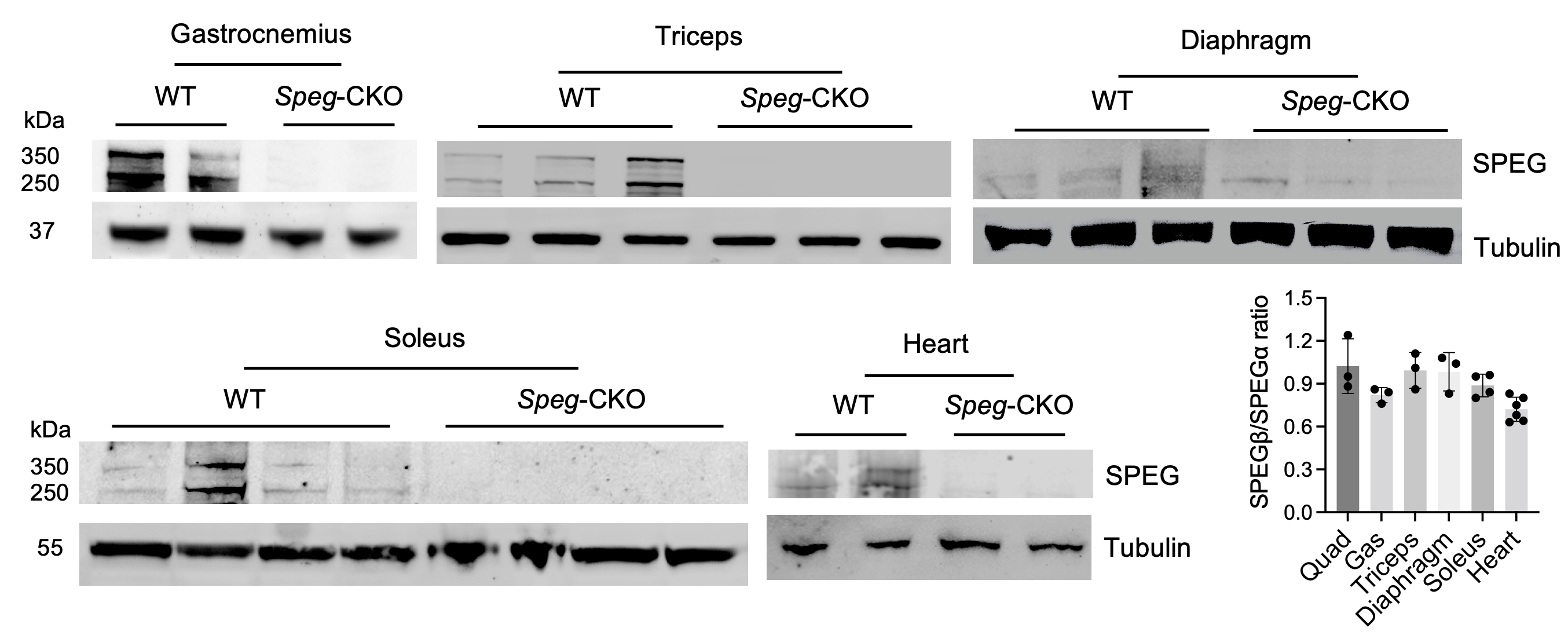
**

**Figure S1. Immunoblot analysis of SPEG isoform ratio (SPEGβ/ SPEGα) among mouse striated muscles.** Tubulin is used as a loading control. Quad: quadriceps; Gas: gastrocnemius.

**
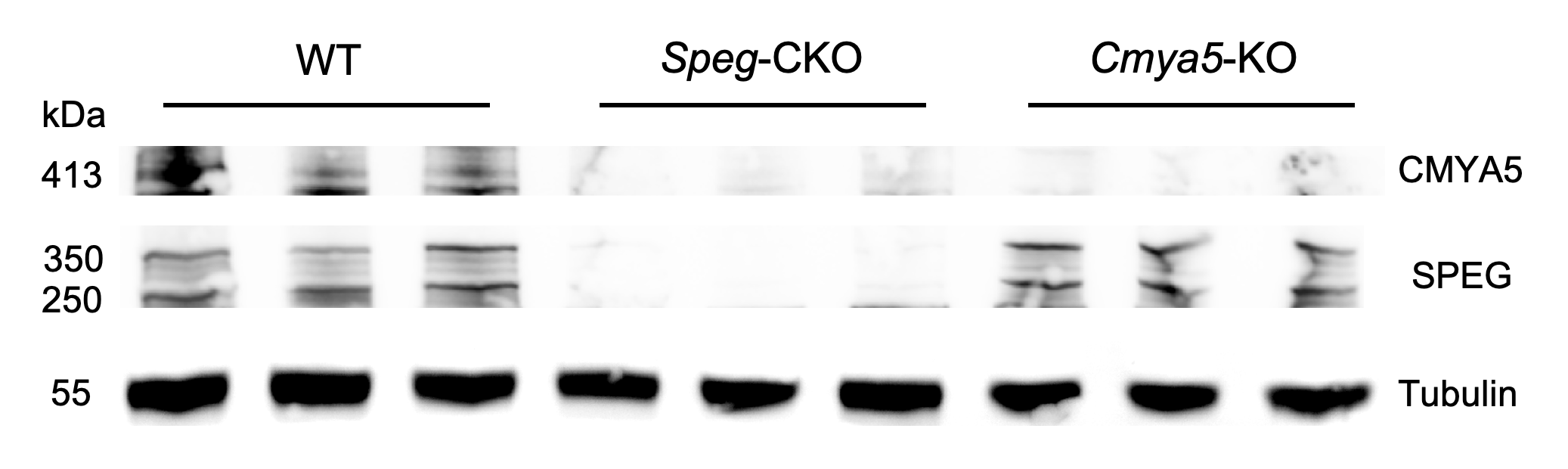
**

**Figure S2. Immunoblot analysis of CMYA5 relative to the expression of tubulin in quadriceps muscle of WT, *Speg*-CKO, and *Cmya5*-KO mice.**

**
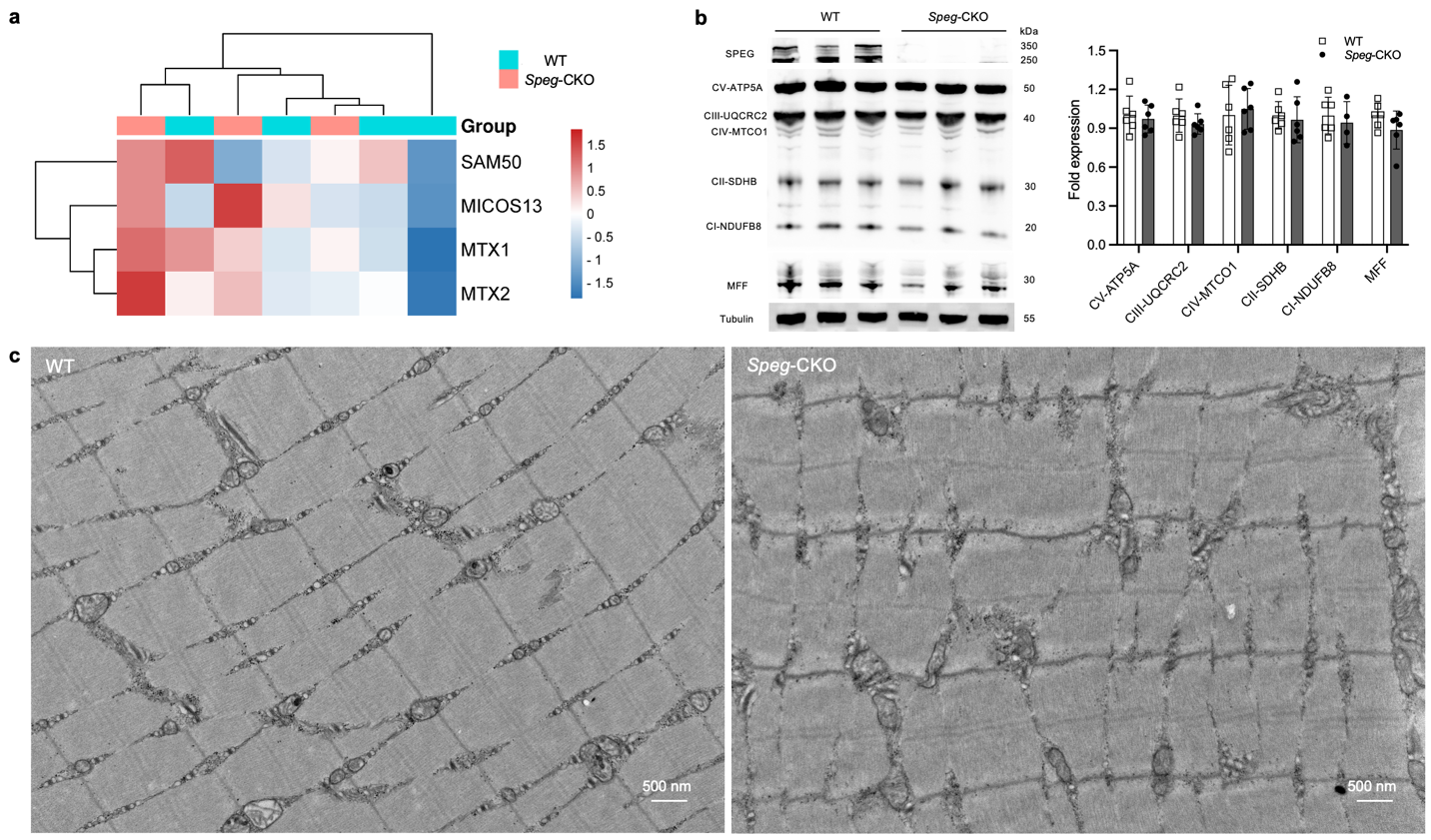
**

**Figure S3. No statistical difference in mitochondria associated proteins between *Speg*-CKO and WT muscles.** (a) Heat map of mitochondrial contact site and cristae organizing system complex proteins detected from proteome data. (b) Immunoblot analysis and quantification of mitochondrial OxPhos (CI subunit NDUFB8, CII subunit SDHB, CIII-Core protein 2 (UQCRC2), CIV subunit I (MTCO1), and CV alpha subunit (ATP5A)) and dynamics proteins (MFF: mitochondrial fission factor) relative to the expression of tubulin in skeletal muscles of WT and *Speg*-CKO mice (n ≥ 4 per genotype). (c) Electron micrographs in quadriceps from WT and *Speg*-CKO mice.

**
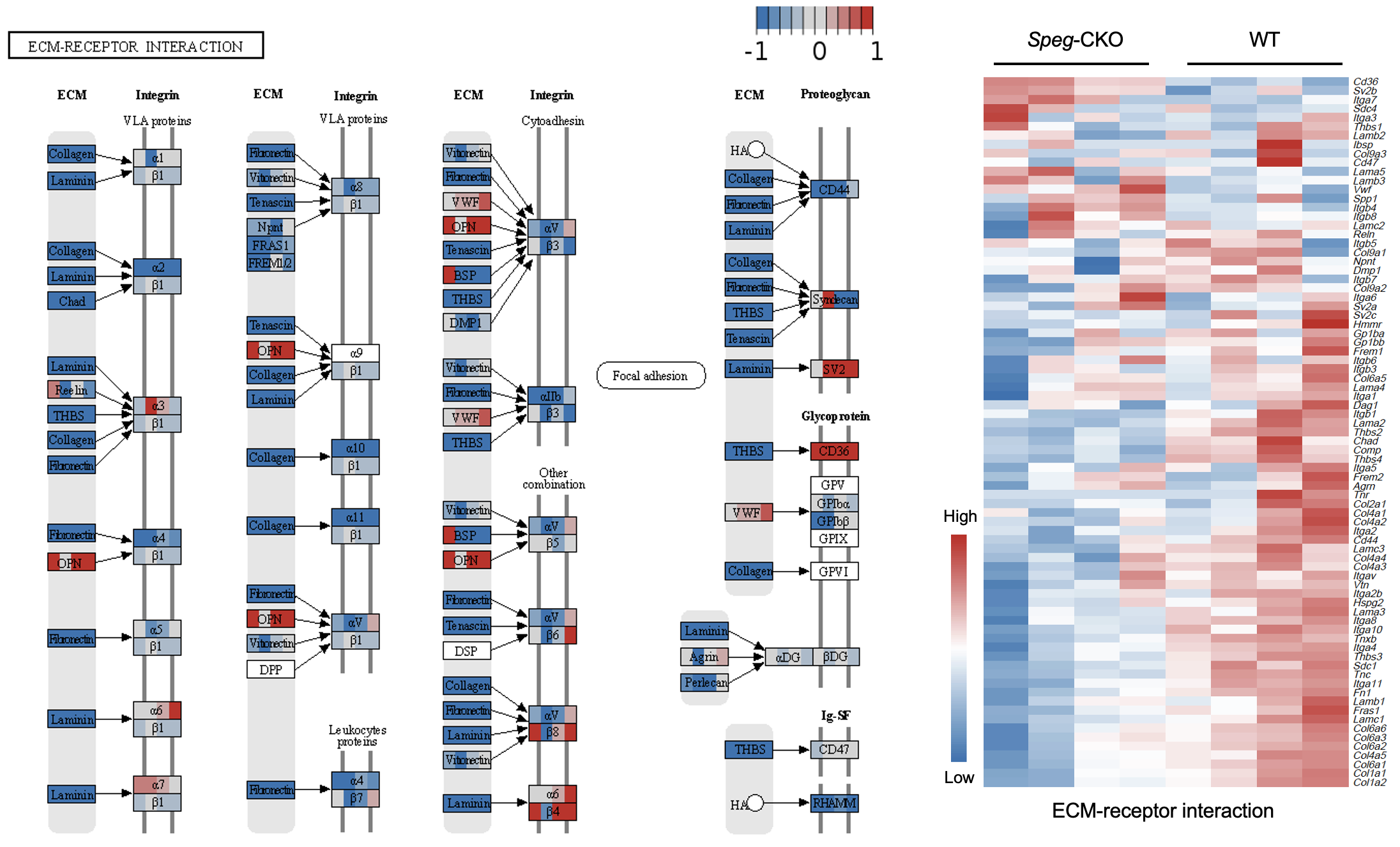
**

**Figure S4. Dysregulated transcripts detected in the pathways of ECM-receptor interaction and heat map of these genes that were detected in the ECM-receptor interaction by transcriptome analysis.**

**
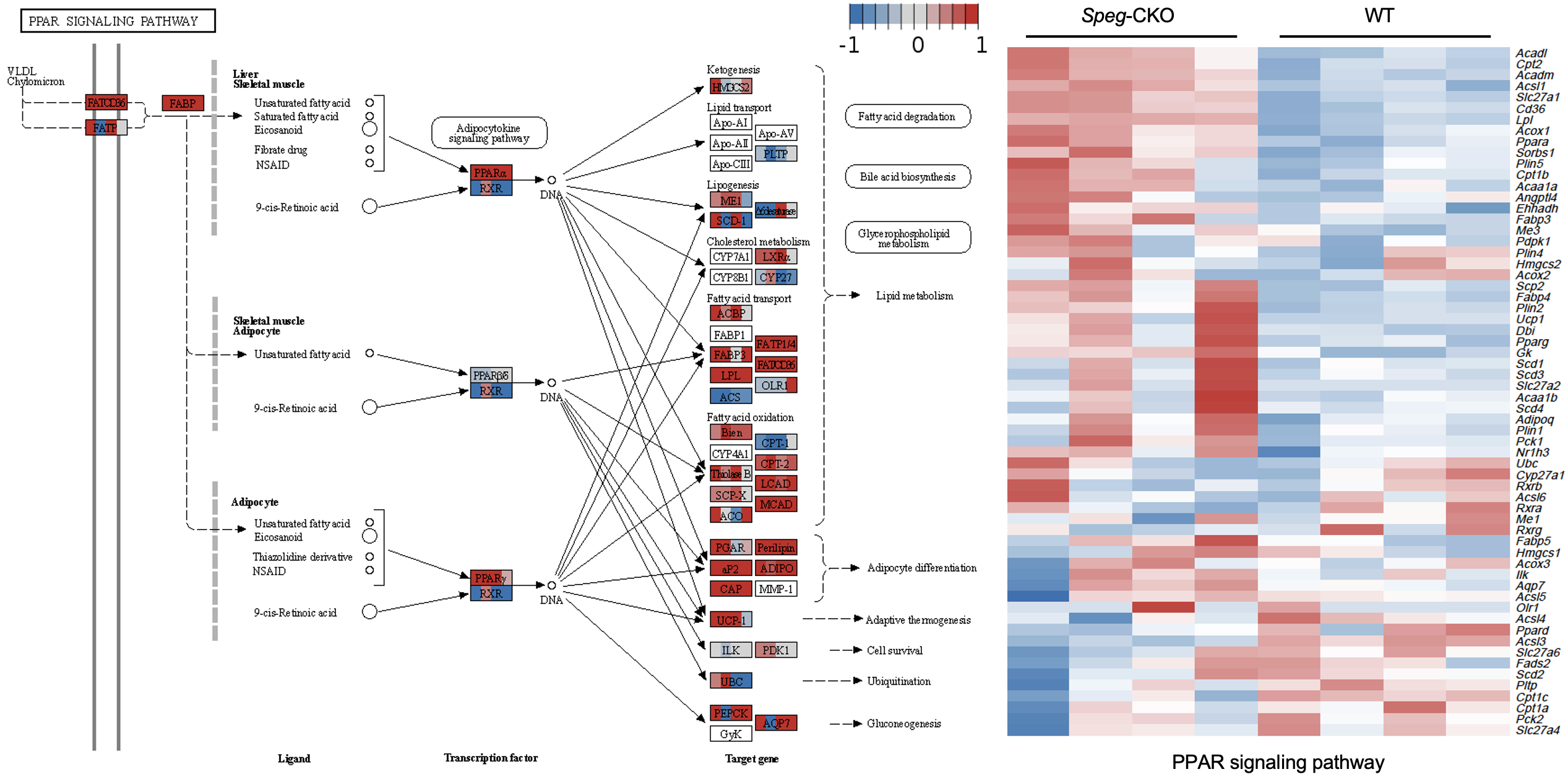
**

**Figure S5. Dysregulated transcripts detected in the pathways of peroxisome proliferator-activated receptors signaling and heat map of these genes that were detected in the PPAR signaling pathways by transcriptome analysis.**

**
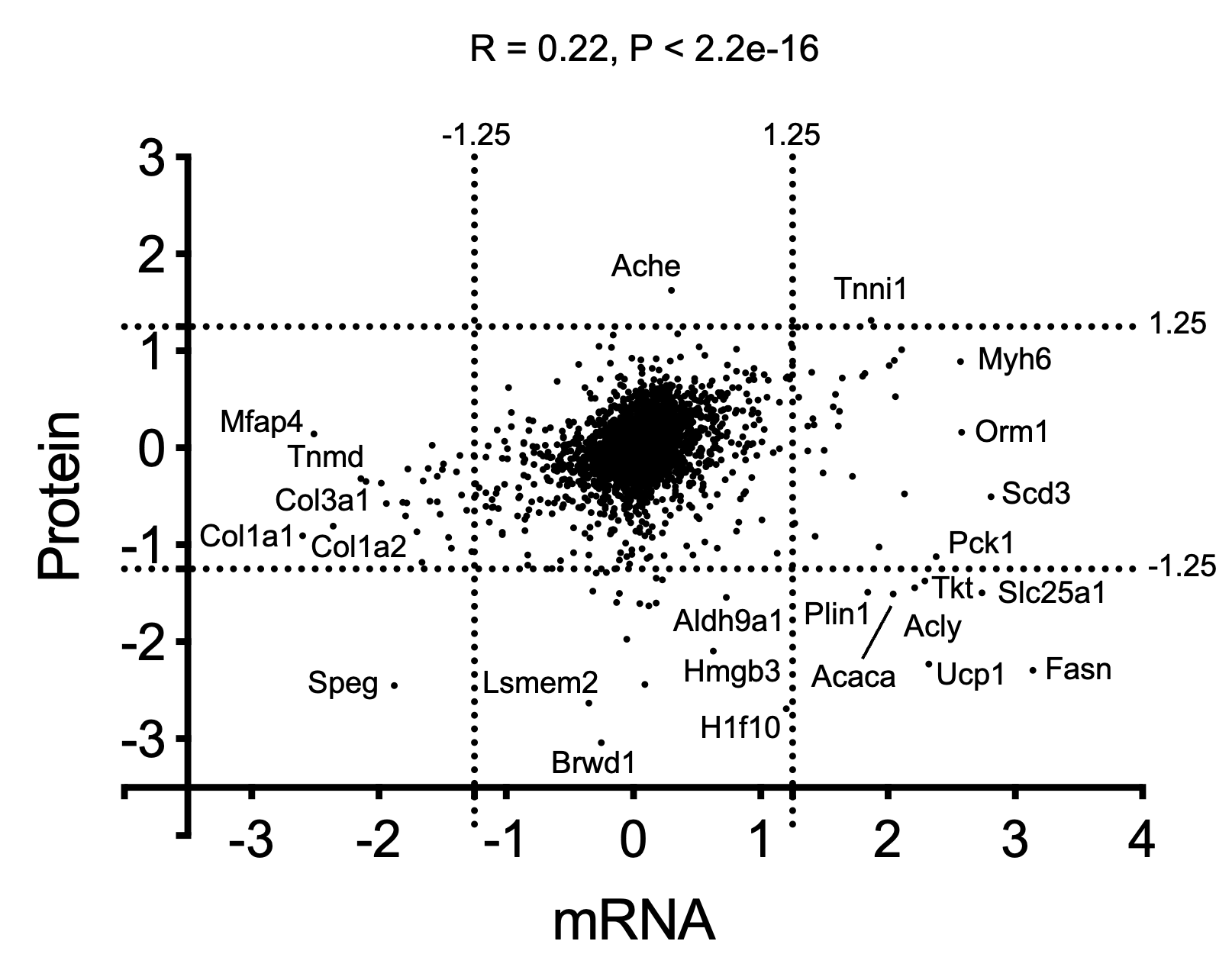
**

**Figure S6. No strong correlation was found in the Pearson correlation analysis of proteomic and transcriptome data.** Pearson correlation between mRNA and protein levels measured by RNASeq and mass spectrometry using skeletal muscles of WT and *Speg*-CKO mice.

**Figure S7. Real-time quantitative PCR (qRT-PCR) analysis for genes of interest using mRNA extracted from mouse skeletal muscle**. *Actb* as the reference gene for normalization (*P < 0.05, n = 4 per group; unpaired 2-tailed t test). qRT-PCR primers were listed in **Table S6.**

**References**

1. Agrawal, P.B., et al., *Normal myofibrillar development followed by progressive sarcomeric disruption with actin accumulations in a mouse Cfl2 knockout demonstrates requirement of cofilin-2 for muscle maintenance.* Hum Mol Genet, 2012. **21**(10): p. 2341-56.

2. Shevchenko, A., et al., *Mass spectrometric sequencing of proteins from silver-stained polyacrylamide gels.* Analytical chemistry, 1996. **68**(5): p. 850-858.

3. Peng, J. and S.P. Gygi, *Proteomics: the move to mixtures.* Journal of mass spectrometry, 2001. **36**(10): p. 1083-1091.

4. Eng, J.K., A.L. McCormack, and J.R. Yates, *An approach to correlate tandem mass spectral data of peptides with amino acid sequences in a protein database.* Journal of the american society for mass spectrometry, 1994. **5**(11): p. 976-989.

5. Doellinger, J., et al., *Sample preparation by easy extraction and digestion (SPEED)-a universal, rapid, and detergent-free protocol for proteomics based on acid extraction.* Molecular & Cellular Proteomics, 2020. **19**(1): p. 209-222.

6. Elias, J.E. and S.P. Gygi, *Target-decoy search strategy for increased confidence in large-scale protein identifications by mass spectrometry.* Nature methods, 2007. **4**(3): p. 207-214.

7. Rad, R., et al., *Improved monoisotopic mass estimation for deeper proteome coverage.* Journal of Proteome Research, 2020. **20**(1): p. 591-598.

8. Eng, J.K., T.A. Jahan, and M.R. Hoopmann, *Comet: an open‐source MS/MS sequence database search tool.* Proteomics, 2013. **13**(1): p. 22-24.

9. Huttlin, E.L., et al., *A tissue-specific atlas of mouse protein phosphorylation and expression.* Cell, 2010. **143**(7): p. 1174-1189.

10. Beausoleil, S.A., et al., *A probability-based approach for high-throughput protein phosphorylation analysis and site localization.* Nature biotechnology, 2006. **24**(10): p. 1285-1292.

11. Schweppe, D.K., et al., *Full-featured, real-time database searching platform enables fast and accurate multiplexed quantitative proteomics.* Journal of proteome research, 2020. **19**(5): p. 2026-2034.

12. Schweppe, D.K., et al., *Characterization and optimization of multiplexed quantitative analyses using high-field asymmetric-waveform ion mobility mass spectrometry.* Analytical chemistry, 2019. **91**(6): p. 4010-4016.
